# Intravital Imaging of a Massive Lymphocyte Response in the Cortical Dura of Mice after Peripheral Infection by Trypanosomes

**DOI:** 10.1371/journal.pntd.0003714

**Published:** 2015-04-16

**Authors:** Jonathan A. Coles, Elmarie Myburgh, Ryan Ritchie, Alana Hamilton, Jean Rodgers, Jeremy C. Mottram, Michael P. Barrett, James M. Brewer

**Affiliations:** 1 Wellcome Trust Centre for Molecular Parasitology, University of Glasgow, Glasgow, United Kingdom; 2 Institute of Infection, Immunity and Inflammation, College of Medical, Veterinary and Life Sciences, University of Glasgow, Glasgow, United Kingdom; New York University School of Medicine, UNITED STATES

## Abstract

Peripheral infection by *Trypanosoma brucei*, the protozoan responsible for sleeping sickness, activates lymphocytes, and, at later stages, causes meningoencephalitis. We have videoed the cortical meninges and superficial parenchyma of C56BL/6 reporter mice infected with *T*.*b*.*brucei*. By use of a two-photon microscope to image through the thinned skull, the integrity of the tissues was maintained. We observed a 47-fold increase in CD2+ T cells in the meninges by 12 days post infection (dpi). CD11c+ dendritic cells also increased, and extravascular trypanosomes, made visible either by expression of a fluorescent protein, or by intravenous injection of furamidine, appeared. The likelihood that invasion will spread from the meninges to the parenchyma will depend strongly on whether the trypanosomes are below the arachnoid membrane, or above it, in the dura. Making use of optical signals from the skull bone, blood vessels and dural cells, we conclude that up to 40 dpi, the extravascular trypanosomes were essentially confined to the dura, as were the great majority of the T cells. Inhibition of T cell activation by intraperitoneal injection of abatacept reduced the numbers of meningeal T cells at 12 dpi and their mean speed fell from 11.64 ± 0.34 μm/min (mean ± SEM) to 5.2 ± 1.2 μm/min (p = 0.007). The T cells occasionally made contact lasting tens of minutes with dendritic cells, indicative of antigen presentation. The population and motility of the trypanosomes tended to decline after about 30 dpi. We suggest that the lymphocyte infiltration of the meninges may later contribute to encephalitis, but have no evidence that the dural trypanosomes invade the parenchyma.

## Author Summary

African trypanosomes are motile parasites that cause sleeping sickness. They multiply first in the blood then cause death mainly by effects on the brain: immune system cells, including T cells and dendritic cells, play major roles in this. Thinking we might see the attack on the brain, we infected mice with trypanosomes and used a two-photon microscope, which allowed us to image the superficial brain and the delicate tissue between the skull and the brain called the meninges without making a hole in the skull. The mice (which were anesthetized) had been genetically modified so that T cells and dendritic cells were fluorescent, as were the trypanosomes. We did not notice much happening in the brain itself, but in the meninges, in a compartment called the dura, huge numbers of T cells and dendritic cells appeared. Trypanosomes also moved from the blood into this compartment. Since T cells, dendritic cells and trypanosomes had not been videoed in the meninges before, we began by observing them carefully: their numbers, their movements and their interactions. The accumulation of lymphocytes is a sign of meningitis, a feature of infection by a wide range of pathogens and our results suggest interesting future work.

## Introduction

Human African trypanosomiasis, or sleeping sickness, results from infection by sub-species of the protozoan *Trypanosoma brucei* and is normally fatal if untreated. At early stages, trypanosomes multiply in the blood and peripheral organs where they are readily killed by available drugs. In the absence of early treatment, trypanosomes can invade the brain parenchyma [1] and for this CNS stage of the disease current treatments are unsatisfactory in that they are logistically difficult to administer, often have severe side effects, and are confronted by the emergence of resistant strains [2–4]. The development of new drugs would be aided by a better understanding of the onset of CNS stage disease. Peripheral lymphocytes are activated in trypanosomiasis [5–8], and the immune system and cytokines are central to the neuropathology [9–13]. An aspect of this immune response, infiltration of the meninges by leukocytes, was reported by Mott [14] and is frequently referred to in review articles (e.g.,[10, 11, 13]), but descriptions in research articles seem to be scanty. Most experimental work on animal models of meningoencephalitis caused by trypanosomes (or by other pathogens) has depended on analysis of homogenized tissue (e.g. [15]), histological sections (e.g., [16, 17]) or cerebrospinal fluid (e.g. [16]). In the present work, we have profited from the development of two-photon microscopy, which allows in vivo imaging with micron resolution through the thinned skull of the mouse [18–20]. In this way, moving cells in the meninges and superficial cortex can be videoed while the integrity of the delicate tissue is preserved. T cells have previously been videoed in the exposed spinal meninges in experimental autoimmune encephalopathy [21, 22], and in the cortical meninges after occlusion of the middle cerebral artery [19], but in the latter case the location within the meninges was not determined. We have briefly reported that in CD-1 mice infected with *T*.*b*. *brucei* GVR35, a standard model of sleeping sickness, trypanosomes invade the meninges [20]. In order to image T cells and dendritic cells (as well as trypanosomes) we have now used genetically modified C57BL/6 mice, and followed the progression of meningitis up to 40 days post infection (dpi).

The question of where in the meninges the lymphocytes and trypanosomes are located would seem to be very relevant to the role, if any, of these actors in the development of CNS pathology. The meninges comprise two main compartments: the dura, and, below it, the leptomeninges (subarachnoid space and pia mater), which are separated from the dura by the apparently impermeable arachnoid membrane [23–25]. The subarachnoid space contains CSF and is continuous with the perivascular spaces of vessels penetrating the neural brain [26]. Hence, lymphocytes and pathogens in the subarachnoid space should face little obstacle to movement into the neural brain [21], while any in the dura would be relatively isolated from it. It is to be noted that in small mammals the subarachnoid space, except where it accommodates the surface pial vessels, is shallow or occluded over the cortical convexities [24, 27, 28]. The dural space [23] is vascularized by ramifications of the meningeal arteries, and richly innervated by the trigeminal system [29–31]. Studies on migraine have demonstrated that the dura is an interface between the nervous and immune systems [32, 33].

It is known that in two other murine models of disease, experimental autoimmune encephalomyelitis and middle cerebral artery occlusion, there is an increase in T cells outside pial vessels [19, 21]. In contrast, we find that, in our infection model, the great majority of the meningeal T cells, dendritic cells and trypanosomes are in the dura. We have analyzed their numbers, movements, and interactions. Since meningoencephalitis is a common outcome of infection by many pathogens, including bacteria, viruses, protozoa, fungi and cancer, and in autoimmune diseases such as multiple sclerosis, the observations may also be relevant to diseases other than trypanosomiasis.

## Materials and Methods

### Ethics statement

All animal experiments were performed in accordance with the Animals (Scientific Procedures) Act 1986 and the University of Glasgow care and maintenance guidelines. All animal protocols and procedures were approved by The Home Office of the UK government and the University of Glasgow Ethics Committee. Specifically, the number of animals was kept to a minimum and all surgery and imaging were done under terminal anesthesia.

### Generation of expression constructs

The fluorescent protein genes, *EGFP*, *mCherry*, *mKate2* (Evrogen) and *tdTomato* (Clontech) were amplified using primers to add *HindIII* and *BamHI* sites and cloned into pGEMT. The *HindIII*/*BamHI* digested genes were each cloned into pHD1034 (from C. Clayton, [34]) to generate pHD1034-EGFP (pGL2179), pHD1034-mCherry (pGL2160), pHD1034-*mKate2* (pGL2174) and pHD1034-tdTomato (pGL2221).

#### Generation and culturing of fluorescent *T*. *brucei*

Culture-adapted *Trypanosoma brucei* strain GVR35 bloodstream forms [20] were grown *in vitro* at 37°C, 5% CO_2_ in Iscove's Modified Dulbecco's Medium (Gibco) supplemented with 20% heat-inactivated fetal calf serum (PAA), 20% Serum Plus, 0.75 mM hypoxanthine in 0.1 N NaOH, 4.1 mM glucose, 0.12 mM thymidine, 1.5 mM sodium pyruvate, 0.037 mM bathocuproine disulfonic acid, 0.2 mM β-mercaptoethanol, 1.1 mM L-cysteine, 0.38 mM adenosine, 0.38 mM guanosine, 0.83 g.L^-1^ methylcellulose, 0.04 mM kanamycin, 75 units.mL^-1^ penicillin and 0.075 mg.mL^-1^ streptomycin (all Sigma-Aldrich). All genetic modifications were done on culture-adapted GVR35 WT cells less than a week after thawing.

For generation of GVR35 lines expressing fluorescent proteins, 20 μg of *NotI*-linearized plasmid (pHD1034-EGFP, pHD1034-mCherry, or pHD1034-tdTomato) was transfected into 3 x 10^7^ mid-log GVR35 WT trypanosomes using the Human T cell Solution and Amaxa Nucleofector (Lonza) set on program X-001. After recovery for 24 hours, transformed clones were selected by limiting dilution in the presence of 15 μg.mL^-1^ puromycin (Calbiochem). For each fluorescent protein six clones were observed using live epifluorescence microscopy, and the clones with highest fluorescent protein expression were selected for further *in vivo* tests. Stability of protein expression for each clone was confirmed by epifluorescence microscopy of live FP trypanosomes in blood films prepared from mice infected for 20–35 days.

### Mouse infections

C57BL/6 mice and C57BL/6 GM reporter mice were bred and maintained under specific pathogen-free conditions. Mice expressing EGFP or DsRed in T cells under control of the hCD2 promoter were bred from progenitors kindly given by D. Kioussis and A. Patel [35]. Mice expressing EYFP under control of the CD11c promoter (in dendritic cells) are described in [36]. We also used crosses: hCD2-DsRed x CD11c-EYFP. Adult mice (19–30 g body weight) of either sex were infected with 3 x 10^4^ *T*. *b*. *brucei* strain GVR35 (WT or FP) trypanosomes by intraperitoneal injection and monitored for parasitemia by counting trypanosomes in blood taken from the tail vein, using a haemocytometer with a detection limit of 2x10^4^ parasites/mL. The very rare mice that showed motor abnormalities were culled. No differences in the parameters measured were observed between male and female C57BL/6 mice great enough to show up among the scatter of the data. Use of CD-1 mice (for which we had no reporter strains) and *T*.*b*.*brucei* Lister 427 (which is normally lethal by 4 dpi) is described in ref.[20]. Inhibition of T cell activation was studied by injecting abatacept i.p. at 10 mg/kg body weight on alternate days. The abatacept (Orencia), was a gift from Bristol-Meyers Squibb.

### Surgical procedures

#### In vivo imaging

Trypanosomiasis appeared to modify the mouse's response to anesthetics. The procedure adopted was to use a low dose of Hypnorm/Hypnovel (Vetapharm and Roche; 5–7 mL/kg body weight, i.p.) and then place the mouse on a base plate equipped with an anesthetic mask (S1 and S2 Figs), anesthesia being reinforced as necessary with isofluorane in oxygen. The core temperature was maintained at 36.8–37.2°C by a 25 Ω heating mat (De-Icers (MHG) Ltd, Cheltenham, UK) controlled by a rectal probe (50-7221f, Harvard Apparatus). The left parietal skull was exposed and cleaned and the desired center of the imaging area was marked with a fiber-tip pen: the co-ordinates with respect to bregma were 1.8–2.1 mm lateral, 1.8–2.2 mm posterior. A thin stainless steel skull plate with a hole 5 mm in diameter was glued to the skull with a dental adhesive (RelyX Unicem Clicker, 3M ESPE, Seefeld, Germany). The tail was warmed in water and a vein was injected with one or more fluorescent markers (see below). The skull plate was then clamped to holders on the base plate (S1 Fig). Surrounding the hole in the skull plate was a low wall of epoxy adhesive so that the exposed skull could be constantly flushed (S2 Fig). We used a Tris-Buffered Saline (TBS) containing calcium to facilitate blood clotting (NaCl 150 mM; KCl 2.5 mM; CaCl_2_ 2.0 mM; Tris buffer, pH 7.3, 10 mM). Under a stereomicroscope, the skull within the 5 mm hole was ground away using a spherical diamond dental bur (Diama International, London, UK), until the meningeal blood vessels were clearly visible. The drill was run at 30,000 r.p.m. and contact with skull bone was detected mainly by the sound. To examine the regularity of the bone surface the perfusion flow was temporarily stopped and the fluid level allowed to fall. The thickness of the remaining skull bone, which can be measured by its second harmonic generation (SHG) under the two-photon microscope (S3 Fig) was generally 10–20 μm (see [37]). The base plate with the mouse and attached tubes and wires was transferred without delay to the stage of the two-photon microscope (LSM7 MP, Zeiss, Jena, Germany; S2 Fig). A heater (64–0102, Warner) was inserted in the perfusion inflow and adjusted to maintain the temperature of the solution arriving at the skull at 34–36°C. Imaging started within about 20 min of the beginning of the skull thinning and continued for up to 4h. After the imaging, the mouse was euthanized by an overdose of anesthetic or, in some cases, perfused and the brain sliced.

#### Brain slices

The mouse was gravity perfused through the left ventricle with 20 mL phosphate-buffered saline (PBS) containing 10 mM glucose followed by 10 mL glucose PBS containing 30–40 μL of carboxyl quantum dots (525 nm or 655 nm, Invitrogen) to label blood vessels. Alternatively, the DiI labeling solution described by Li et al. [38] was used. 1 mm coronal slices of brain were cut using a brain matrix (Zivic Instruments). Slices were imaged in a temperature controlled chamber (TC344B, Warner) perfused with TBS containing 10 mM glucose.

#### Infusion into the cisterna magna

The mouse's head was tilted forwards and the skin over the occiput region retracted. Infusion was made through a quartz micropipette, tip diameter about 100 μm and length 5 mm, connected to a syringe pump. 10 μL were infused over 10 min (as in [26]).

### Exogenous fluorescent labels

Blood plasma was labeled by injection in a tail vein of a fluorescent marker: 70 kD dextran, conjugated with either fluorescein isothiocyanate ("dextran-FITC") or rhodamineB isothiocyanate ("dextran-rhodamine") both from Sigma, was dissolved at 100 mg/mL PBS and 50–70 μL injected. Alternatively, 20–30 μL of quantum dot solution was used (QTracker, Invitrogen, emission peak at 705, 655, or 625 nm). Furamidine ([2,5-bis(4-amidinophenyl)furan], also known as DB75 [39]) was a gift from David W. Boykin; it was routinely injected with the vascular marker. Furamidine, dissolved in DMSO at 1 mg per 40 μL and injected at a final concentration of 10 mg/kg body weight, labeled nuclei of trypanosomes and also certain host cells (S4 Fig).

### Two-photon microscopy

Excitation light came from a Ti-sapphire femtosecond laser tunable from 700 to 1050 nm (Chameleon Ultra II, Coherent, Santa Clara, USA). To obtain long wavelengths with higher power, the output of the Ti-S laser passed through an optical parametric oscillator (OPO, Coherent): when pumped by the Ti-S laser at about 800 nm, outputs up to 1200 nm were obtained. It was possible to use part of the pump beam simultaneously with the OPO output. The intensity of the Ti-S beam bypassing the OPO was regulated by an acousto-optical modulator controlled by the imaging program (Zen 2010, Zeiss). The intensity of the OPO beam was varied manually by a polariser. The scan head (Zeiss LSM7 MP) had a maximum rate of 8 frames per sec. Almost all the imaging was done with a 20x water immersion objective, NA 1.0. with high NIR transmission (W Plan-Apochromat, Zeiss). Excitation and emitted light were separated in the microscope nose by a dichroic mirror with a cutoff at 740 nm. Five detectors of non-descanned fluorescence were available, three multialkali photodiodes, and two GaAsP detectors.

#### Intrinsic signals

Skull bone showed little fluorescence but strong SHG (S3 Fig). SHG from collagen was strongest with excitation at about 1100 nm.

### Image analysis

Zen 2010 (Zeiss), Volocity (Perkin-Elmer), Imaris 7.4.1. (Bitplane), and ImageJ (N.I.H) were used. Care was taken to establish the anatomical orientation of the image files, and images were transformed as necessary so that rostral was up and left lateral to the left. In some cases, when signal from dendritic cells or blood vessels leaked into the T cell channel, the contamination was removed by subtracting the unwanted channels using Zen. On Volocity and Imaris, one or more channels of the image were sometimes smoothed, and usually the contrast was enhanced. Observation of fiducial features (such as crossed collagen fibers) showed that tissue drift in the X and Y directions was never more than 5.8 μm/h, and usually undetectable (less than 1 μm/h). In the Z direction there was, on average, significant drift corresponding to increasing distance between the microscope objective and the dura at 4.9 μm/h, SD 4.9 μm/h, *p* < 0.026.

#### Imaging trypanosomes

Extravascular trypanosomes in the meninges moved too fast to be imaged in three dimensions and were therefore imaged in a nominal XY plane. However, since Z resolution was poor (several microns), this allowed for some coverage in the Z direction. Scanning downwards from the skull showed that the great majority of extravascular trypanosomes were captured in one nominal XY plane (usually about 20 μm below the skull). To obtain approximate values for the numbers of trypanosomes per unit area of meninges, we chose areas at random, focused at the depth of maximum trypanosome population and made time series at the maximum scan rate for 100 cycles (giving a total time of 12 s). We then played back the videos at reduced speed and counted all the trypanosomes that appeared. The size of the imaged area was chosen to include fewer than 30 trypanosomes, and was usually 212 μm^2^ or 143 μm^2^. Up to 10 non-overlapping areas were counted in each mouse.

#### Imaging T cells and dendritic cells

To locate these cells, Z-stacks >100 μm deep were acquired. To track T cells, the lower and upper limits of a Z-stack were chosen to include nearly all the T cells in the field. Stacks 12–38 μm deep were acquired at intervals of 8–30 s for 10–45 min. Movements were analyzed with Volocity and/or Imaris. In addition to speed, velocity, and displacement rate, for a selection of T cell tracks we calculated the skewness of the distribution of instantaneous speeds, skewness being defined as (∑(S_i_ - μ)^3^)/Ns^3^ where S_i_ is the speed over time interval *i*, μ is the mean speed of that cell, N is the number of intervals in the track and s is the standard deviation of the speed distribution [40]. We selected non-stationary tracks (with a mean speed > 1 μm/min) and calculated the skewness of the speed distribution for at least 10 tracks per mouse ≥ 11 dpi.

### Statistics

Student's *t* test was used for comparisons between mice (log (mean number of cells), mean speed of cells, etc.) and the Mann-Whitney test for distributions that were not normal (individual cell speeds, velocities, etc).

## Results

**Fig 1:**
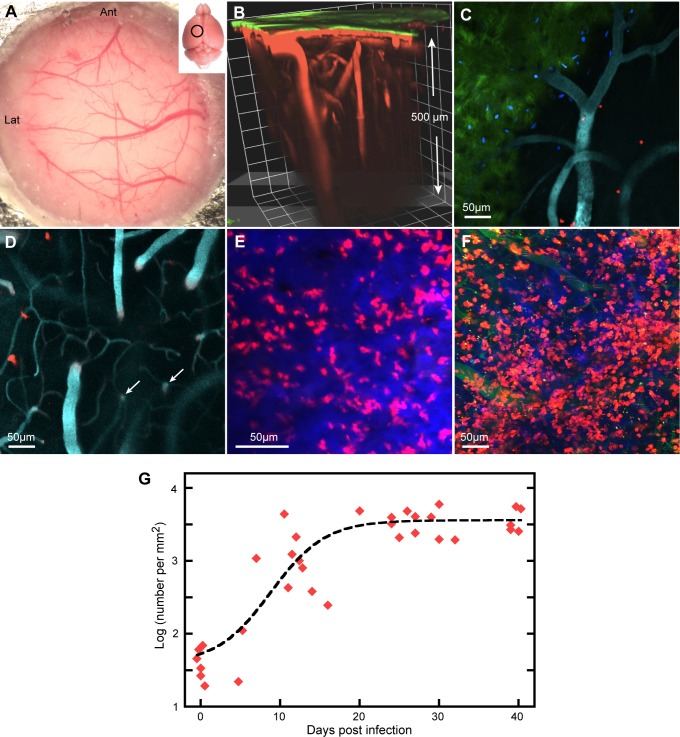
Peripheral infection increases the number of T cells in the meninges. **A**. The thinned skull, showing parts of the metal plate surrounding the 5 mm hole. Generally, arteries arrive from the left, veins leave to the right. Inset: the approximate position of the area imaged. **B.** 3D reconstruction from a Z-stack showing the skull (green) and larger blood vessels, horizontal in the meninges and vertical in the brain parenchyma. **C.** Z-projection 30 μm thick in an uninfected mouse showing sparse CD2^+^ cells (DsRed) close to the skull. The skull is slightly inclined to the XY plane and is seen in green to the lower left. Cell nuclei labeled by i.v. injection of furamidine are blue. Blood vessels (cyan) are labeled with quantum dots. **D**. At deeper levels, surface vessels turn downwards and vertical vessels in the parenchyma are visible (arrows). Three T cells are at about the level of the horizontal vessel. This is another 20 μm Z-projection of the same XY field as (**C**). **E,F.** Deep Z-projections (143 μm and 188 μm respectively) at 7 dpi (**E**) and 39 dpi (**F**), showing increased numbers of T cells. The blue in (**E)** is from the skull. Blood vessels in (**F**) are green**. G**. Numbers per mm^2^ of meningeal T cells plotted on a log scale against dpi. Each symbol corresponds to one mouse. The dashed line is a fitted sigmoid.

Optical access to the parietal cortex was gained by thinning the skull (Fig 1A) and imaging was started within about 30 min after the beginning of skull surgery. Skull bone was visualized by its second harmonic signal (SHG, Fig 1B and S3 Fig) and blood vessels by a blood marker (red in Fig 1B). With averaging of scans, it was possible to resolve large blood vessels to a depth of 400 μm (Fig 1B); objects, that were smaller and moving, such as T cells, could be detected down to about 150 μm below the skull. All quantitative data are from C67BL/6 mice infected with *T*.*b*.*brucei* GVR35, with some supporting illustrations from other strains of mice or *T*.*b*.*brucei*.

### Infection causes an increase in the number of T cells in the cortical meninges

In uninfected reporter mice, small numbers of T cells expressing fluorescent protein (FP) under control of the CD2 promoter were visible at the level of horizontal meningeal vessels (Fig 1C) but were very rare in the parenchyma where the larger blood vessels are vertical and linked by characteristic sinuous capillaries (Fig 1D). After intraperitoneal injection of trypanosomes, the number of T cells in the meninges appeared to increase by 7 dpi (Fig 1E) and increased further (Fig 1F, 22 dpi). To count T cells in an image field, we acquired a Z-stack deep enough to include the upper and approximate lower limits of the population. Several random fields were imaged in each mouse and the mean number of T cells per unit area of meninges calculated. A significant increase was observed by 11 dpi (*p* = 0.001), the increase leveled off at about 20 dpi and was maintained to 40 dpi, the longest we maintained infected mice (Fig 1G).

### Infection causes an increase in the number of meningeal dendritic cells

**Fig 2:**
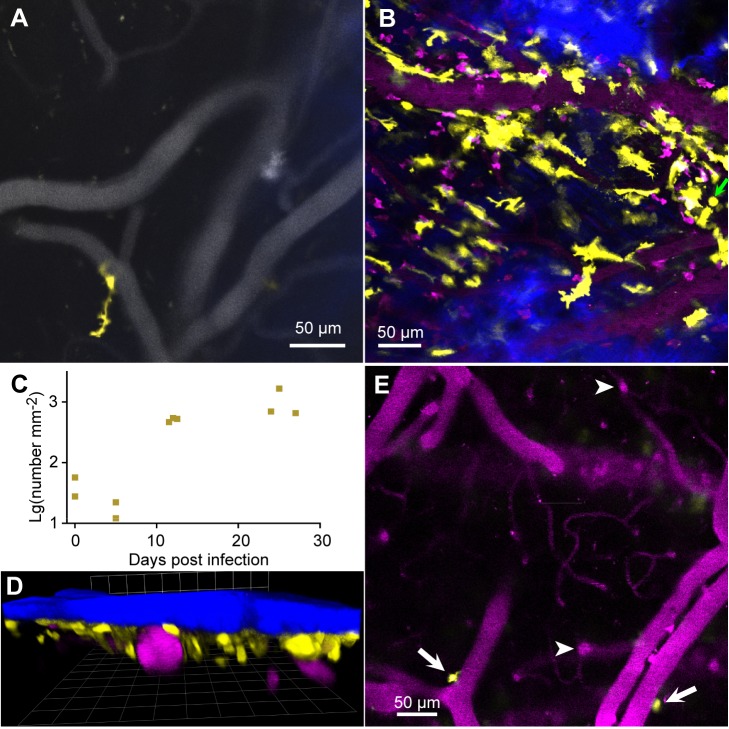
Infection increases the number of dendritic cells in the meninges. **A.** In uninfected mice, there was a small population of CD11c^+^ (EYFP) cells (yellow) in the meninges, mainly close to dural vessels (gray). **B**. The numbers increased by 12 dpi. SHG signal (blue) indicates the proximity of the skull; blood vessels are magenta. **C**. Approximate numbers of dendritic cells per unit area of meninges. Each symbol is the mean for one mouse. **D**. In a 3D reconstruction of a 139 μm Z-stack at 27 dpi, the dendritic cells are seen to be close under the skull (blue) and above or beside vessels (magenta). The grid spacing is 42.4 μm **E**. At 11 dpi in the same mouse as (**B**) a deeper XY plane shows only two CD11c^+^(YFP) cells, located on horizontal pial vessels embedded in the brain surface (arrows). Arrowheads point to vertical vessels below the pia mater.

T cells are dependent on signals from antigen-presenting cells, notably dendritic cells, for activation, and dendritic cells are present in the normal murine dura [28, 41]. We imaged reporter mice expressing Enhanced Yellow Fluorescent Protein (EYFP) under control of the CD11c promoter so that myeloid dendritic cells and a small sub-population of macrophages were fluorescent [36]. In uninfected mice, small numbers of CD11c^+^(EYFP) cells were present, located on meningeal vessels (Fig 2A). Most had the irregular shape and constant extension and retraction of processes that identified them as dendritic cells (Fig 2A and S1 Video). A few CD11c^+^ cells were spherical and usually not adjacent to blood vessels (not shown).

The numbers of CD11c^+^ cells increased markedly by 12 dpi (Fig 2B). The great majority of them were irregularly shaped and many were velate. These morphologies made it difficult to count them with any accuracy, but we estimate that infection increased their numbers by a factor of at least ten (Fig 2C). Nearly all were within 40 μm of the skull, apart from a minority close to pial veins (Fig 2D). None was observed below the pia mater (Fig 2E).

### The increase in lymphocytes is followed by the appearance of extravascular trypanosomes

**Fig 3:**
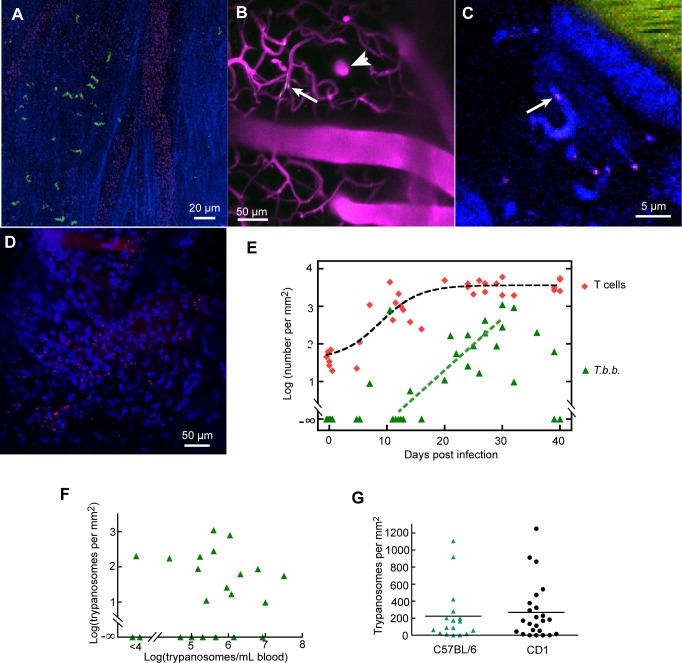
Extravascular trypanosomes appear in the meninges. **A.** GFP trypanosomes in the meninges at 32 dpi. Blue: collagen. Faint magenta: blood marker (Qdots). **B**. At the level of vertical penetrating vessels and parenchymal capillaries, intravascular trypanosomes were detected (arrow), but extravascular trypanosomes were not. 16 dpi. **C**. A trypanosome labeled by intravenous injection of furamidine. In this case, the nucleus gave red fluorescence (arrow) and the cytoplasm gave blue fluorescence. **D**. Trypanosomes (identifiable by their rapid movement as in S5 Video) giving red fluorescence after labeling with i.v. furamidine. Furamidine also produced blue fluorescence from endogenous nuclei. Single plane, 25 dpi, CD-1 mouse. **E**. Numbers of trypanosomes in the meninges as a function of dpi (green triangles). Their appearance tends to be later than the increase in T cells (red lozenges). In 8 mice, T cell number had increased but no trypanosomes were observed in the meninges. **F.** The number of trypanosomes in the dura shows no dependence on paristemia. **G**. Numbers of trypanosomes in the dura 20–40 dpi in C57BL/6 mice and CD1 mice (from [20]).

In mice infected with *T*.*b*.*brucei* GVR35 expressing a fluorescent protein (FP), such as EGFP or tdTomato, extravascular (as well as intravascular) trypanosomes were observed in the meninges, usually from about 11 dpi (Fig 3A and S2 Video). In contrast, in the superficial parenchyma only intravascular ones were observed (Fig 3B), in agreement with other reports that extravascular trypanosomes are rare in the sub-pial cortex of Murinae [17, 20, 42, 43]. Rare extravascular trypanosomes were observed in ventral areas of brain slices (S3 Video). Since the extravascular trypanosomes moved rapidly, they could not be counted without imaging rapidly, so we scanned in one nominal XY plane at the maximum rate of 8 f.p.s. (S4 Video). However, in any one image field, trypanosomes were almost all confined within a depth extending about 5 μm thick above and below the plane of maximum density. Given the finite depth of focus, and the up-and-down motion of the trypanosomes (so they crossed this plane), we could make an approximate count by examining videos of 100 or more frames.

In exceptional cases, trypanosomes in the mouse lost expression of FP. To forestall this possibility, we routinely added the DNA-binding drug furamidine [39] to the solution of blood marker that was injected intravenously. Furamidine is taken up by trypanosomes in vitro and can be detected by its fluorescence [39, 44]. Within about 15 min of intravenous injection in infected mice (10 mg/kg), trypanosomes in the blood and meninges were brightly labeled ([20] Fig 3C and 3D). The labeling was variable: sometimes only the nucleus and kinetoplast were labeled, giving blue or red fluorescence, and sometimes, as in Fig 3C, the nucleus was red and the cytoplasm blue. In any case, trypanosomes could be identified unambiguously by their movement (S5 Video), which was unaffected by furamidine over the imaging period of up to 3h [20]. By comparison of the signals from trypanosomes expressing FP and from furamidine, it was apparent that all meningeal trypanosomes were labeled within 15 min of the injection of furamidine.

Extravascular trypanosomes were commonly observed in the meninges from about 12 dpi, and their numbers tended to increase with time of infection up to about 30 dpi (Fig 3E). In general, the arrival of trypanosomes was later than the increase in T cells (dashed lines in Fig 3E). After 30 dpi the number of meningeal trypanosomes appeared to fall and, in two mice, no trypanosomes were found 39–40 dpi despite parasitemia of 1.4–8.4 x 10^6^ mL^-1^ (Fig 3E). More generally, there was no correlation between the numbers of trypanosomes in the meninges and the numbers in the blood (Fig 3F), suggesting that trypanosomes do not exchange freely between blood and the extravascular space. From 20 to 40 dpi the number of GVR35 trypanosomes in the parietal meninges of C57BL/6 mice ranged from 0 to 1107 mm^-2^ (Fig 3G; 17 mice); the median (89 mm^-2^) did not differ significantly from that in CD-1 mice (Fig 3G, [20]).

We considered the possibility that extravascular trypanosomes observed in the meninges ≈ 0.3–4h after we began to thin the skull had somehow arrived there as an artifactual result of the surgery. Previously, we had reported that, consistently, no extravascular trypanosomes were found in the meninges three days after infection with *T*.*b*.*brucei* Lister 427, despite very high parasitemia [20]. This result is supported by the present experiments, in which, in a further nine mice with parasitemia, no extravascular trypanosomes were found in the meninges (Fig 3F). When trypanosomes were present, there was no correlation between meningeal population and parasitemia (Fig 3F). These results argue strongly against a rapid arrival of extravascular trypanosomes during the surgery or the first minutes after it. Nor did surgery initiate a significant arrival that was more gradual, since the number of meningeal trypanosomes did not noticeably increase during up to 3 hours of imaging.

### The locations of trypanosomes and lymphocytes within the meninges

**Fig 4:**
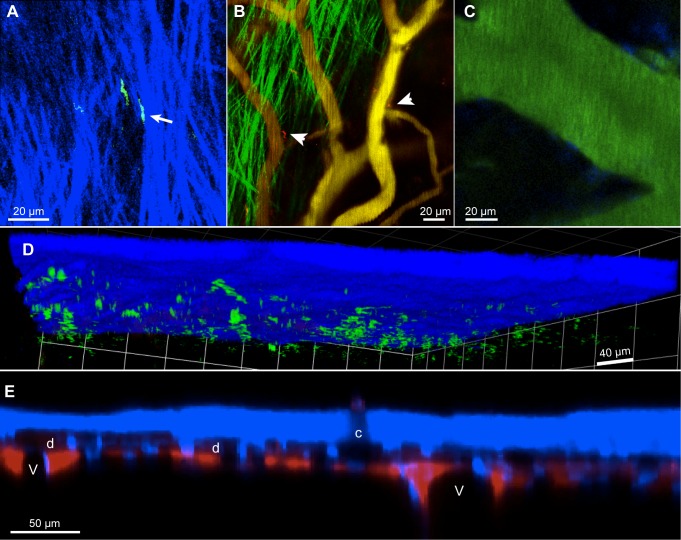
The location of extravascular trypanosomes. **A.** A frame from S6 Video showing GFP trypanosomes in the plane of extracellular collagen. The movement of one trypanosome appeared to constrained by the collagen (arrow). **B**. Trypanosomes (red, arrow heads) were frequently observed outside small, somewhat irregular, vessels at the level of collagen (green, see S7 Video). **C.** Trypanosomes were not observed at the level of large pial vessels. Scale bars **A,B,C,** 20 μm**. D**. In a 3D reconstruction of a 741s Z-stack acquisition of trypanosomes (green), they are seen to be within about 40 μm under the skull. **E**. Texas Red infused in the cisterna magna labeled spaces adjacent to pial veins (V), and patches at that level, leaving a space (d) beneath the skull. Blue signal is from skull bone and furamidine-labeled nuclei. The bit of skull giving no SHG signal (c) may correspond to the site where a blood vessel entered the skull. 16 dpi.

Recent papers have reported extravascular trypanosomes in the cortical pia mater [43] or the superficial cortical parenchyma [45], while T cells in the spinal meninges have been reported in the leptomeninges (arachnoid plus pia mater [21]). Knowing that the meninges were intact, and benefitting from signals from structural elements, we attempted to define the compartments containing the trypanosomes and T cells. The upper boundary of the compartment was clearly the skull, as trypanosomes were observed immediately below it. Often they were in the same plane as extracellular collagen, the main component of the dura mater (Fig 4A and S6 Video). Also at about this depth were cell nuclei labeled blue by intravenous furamidine (Fig 3D). These cells are likely to be the 'mesothelial lining cells' of the dura that are labeled by intravascular aminoacridines [24]. Defining the lower boundary of the compartment occupied by trypanosomes, like that of the dura itself [46, 47], was not so straightforward. Trypanosomes were observed close to small horizontal vessels, often with an irregular trajectory and close to collagen (Fig 4B and S7 Video), but were not observed at the level of larger horizontal vessels just above the parenchyma (Fig 4C). The latter were clearly in the subarachnoid space; the former were probably branches of the meningeal artery in the dura. In images acquired over many seconds, the trypanosomes traced out apparently isolated volumes within about 40 μm of the skull (Fig 4D). To delineate the extent of the subarachnoid space we infused dye (Texas Red) in the cisterna magna. From this site, dye is known to flow along the perivascular spaces of surface arteries and enter the subarachnoid space over the cortex [26, 29, 48]. As shown in Fig 4E, Texas Red, infused in this way, labeled spaces adjacent to large pial blood vessels and a thin, patchy, space extending across the cortex, in general agreement with histological studies on Murinae [27, 31, 41, 46, 47]. The dye is excluded from a space beneath the skull that contains nuclei labeled by furamidine. It is this "dural space", and not the subarachnoid space, that appears to be occupied by trypanosomes. Qualitative observation through the dissecting microscope, and vertical sections from sample Z-stacks, suggested that the distance from the skull to pial vessels increased in infected mice.

**Fig 5:**
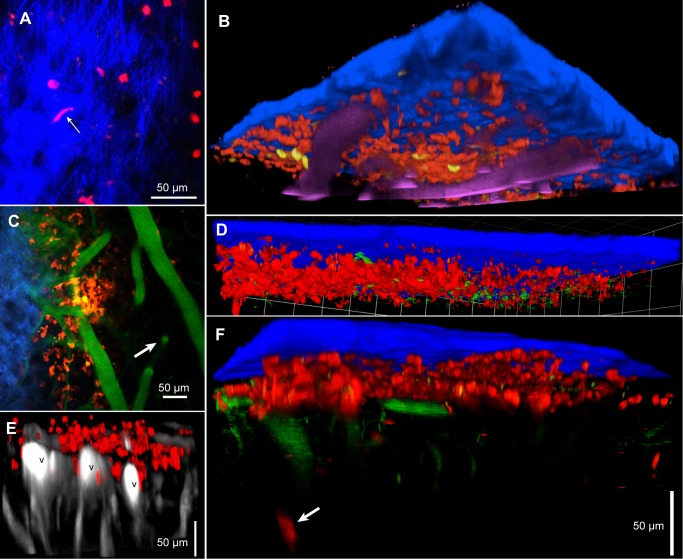
The location of meningeal T cells. **A.** T cells (red) were present at the level of extracellular collagen (blue). One T cell (arrow) can be seen in S8 Video squeezing through collagen. 11 dpi. **B.** T Cells (red) and dendritic cells (yellow) were both mainly above the larger horizontal vessels (magenta). 12 dpi. The imaged area is 424 μm square. **C**. A single plane from the Z-stack of (**B**). The plane is slightly oblique and includes skull or collagen (blue, upper left) and vertical parenchymal vessels (right, arrow, blood marker shown in green). T cells (red) and dendritic cells (yellow) were at the level of dural vessels. **D**. A Z-stack acquired over 741s showing blurred trypanosomes (green) among T cells (red). 11 dpi. The grid spacing is 42.4 μm. **E**. At 30 dpi, T cells are seen adjacent to large horizontal veins. **F**. At 39 dpi, occasional T cells are seen at more than 50 μm below the pia mater (arrow). All scale bars are 50 μm.

Like trypanosomes, T cells were seen close under the skull at the level of extracellular collagen (Fig 5A and S8 Video). At 12 dpi, they were mainly above the horizontal pial vessels, as seen in a 3D reconstruction (Fig 5B) and a slightly oblique single plane (Fig 5C). Dendritic cells were at the same level (Fig 5B and 5C). T cells (at 11 dpi) were seen to be present at the same level as trypanosomes (Fig 5D and S9 Video). However, at 30 dpi, T cells were seen adjacent to large pial veins (Fig 5E) and at 39 dpi, occasional T cells were seen some 50 μm below the pia mater (Fig 5F). In summary, the increase in T cells is greatest in the dura, but T cells can be present at deeper levels.

### T cell movement

**Fig 6:**
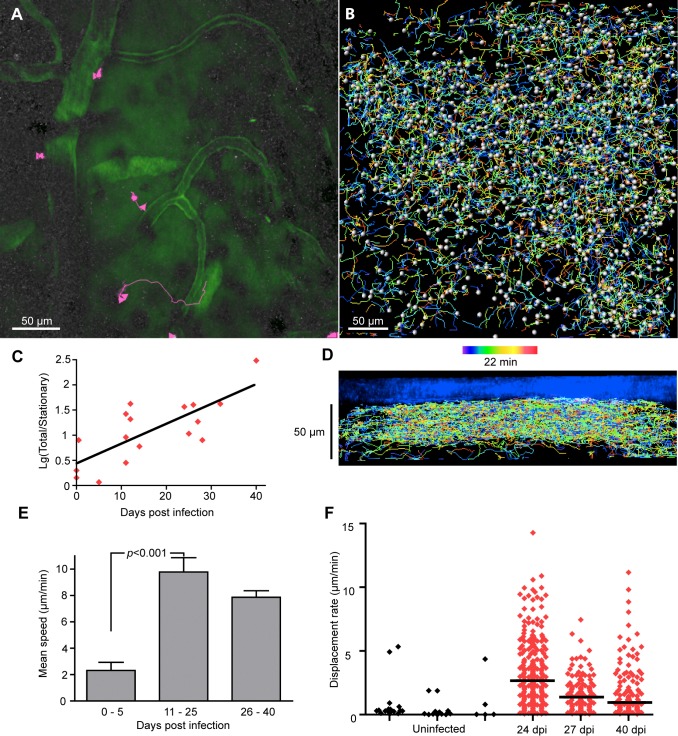
T cell movement in the dura. **A**. Tracks of extravascular CD2^+^ T cells over 25 min in an uninfected mouse (S10 Video). A Z-stack 30 μm deep was acquired. The blood marker was dextran-fluorescein. **B**. Tracks of T cells at 40 dpi in a Z-stack 75 μm thick (S12 Video). Time along tracks is color coded over the 22 min duration of the acquisition, spheres indicate detected cells at the time point indicated (green, about 9 min). **C**. The total number of T cells was divided by the number of stationary T cells (with mean speed < 1 μm/min), and plotted on a log scale as a function of dpi. For the logarithm of this ratio vs dpi, r^2^ = 0.57 and the slope differs from zero with *p* = 0.0005. Each point is for one imaging area in one mouse. **D**. A side view of the tracks in **B** showing that movement was mainly close to horizontal; a few cells moved downwards or upwards at deeper levels. **E.** The mean track speed of each T cell was averaged for each mouse and average values were calculated for groups corresponding to short (0–5 dpi), medium (11–25 dpi) and longer periods of infection (26–30 dpi). Error bars are SEMs, Ns are 4, 6, 4 mice. **F**. (Displacement/track duration) of T cells for durations 300–800 sec in three uninfected mice (black) and three infected mice (red). Horizontal lines show medians.

The activation state of T cells and the nature of their environment are reflected in the speed and pattern of their movement [19, 21, 34, 49–51]. To look for changes in T cell behavior in the cortical meninges during the progression of trypanosomiasis, we made videos, acquiring Z-stacks at 15–30s intervals over periods of up to 45 min. Fig 6A shows typical tracks in an uninfected mouse. Three T cells were almost stationary, one moved rapidly 28 μm along a blood vessel then careered off (S10 Video). In videos from infected mice, a greater proportion of the cells were moving (with speed >1 μm/min; Fig 6B and 6C and S11 Video). Particularly in infected mice, motile cells showed no evident preference for the vicinity of blood vessels.

Nearly all the T cell movements were close to horizontal and within a space about 40 μm deep (Fig 6D). Any net migration (averaged movement) in this plane was small (from zero to four cell diameters per hour) and apparently not in any particular direction, although no migration in the antero-lateral quadrant was observed (S5 Fig). There were occasional vertical movements that extended below this layer; both downward and upward movements were tracked (Fig 6D, S12 Video and S5 Fig), and, on the time scale of the videos (up to 45 min) we detected no net movement downwards towards the parenchyma.

The mean speed of T cells in uninfected mice was 3.4 μm/min, S.D. = 1.4 μm/min, N = 3 mice. By 11–25 dpi this had increased to 9.8 ± 2.6 μm/min (N = 6, Fig 6E). The fastest brief spurt we noticed was at 30.1 μm/min over 40 sec.

Measurement of T cell displacement showed that the T cells were not necessarily confined to small territories, as displacements as great as 93 μm were tracked. Median displacement rate was markedly greater in infected mice compared to uninfected mice, as illustrated by the scatter diagrams from individual mice in Fig 6F.

### Abatacept reduced the lymphocyte response

**Fig 7:**
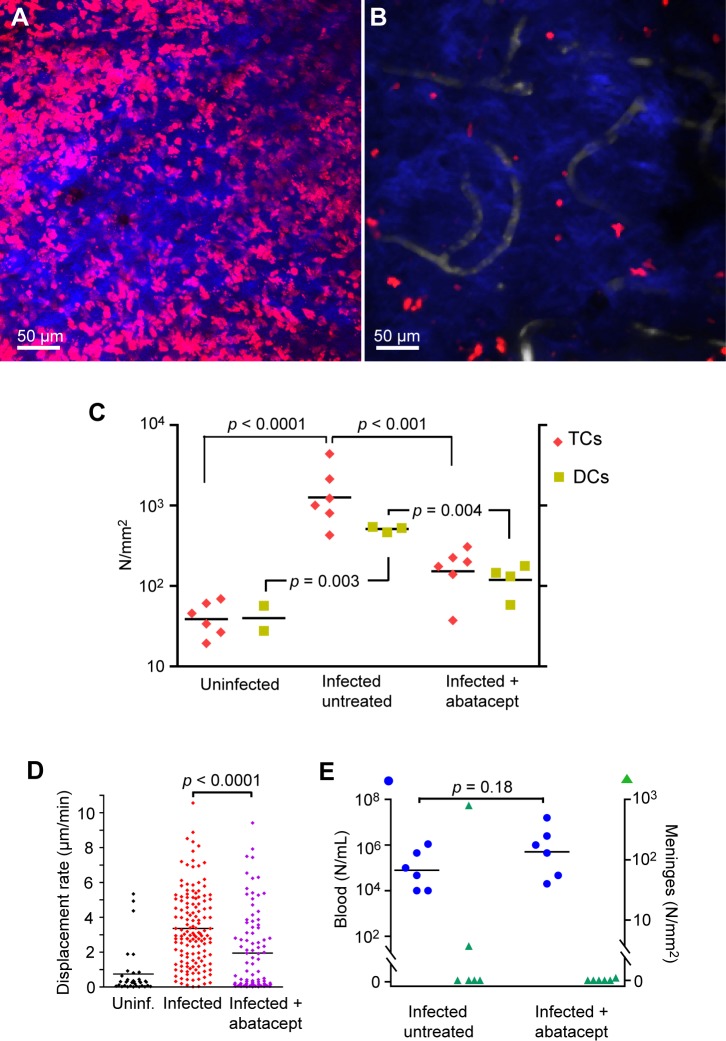
Abatacept reduced the increase in meningeal T cells. **A.** CD2^+^ (DsRed) cells in the meninges at 11 dpi, untreated control. Z-projection, 15 μm. Blue from SHG indicates the proximity of the skull. **B**. A companion mouse treated with abatacept. **C**. Numbers of meningeal T cells and dendritic cells, in uninfected controls, and untreated and treated mice at 11–12 dpi. Each symbol is the mean for several fields for one mouse. *p* values were calculated by Student's *t* test after log transformation. **D**. Mean speeds of T cells in three untreated and three treated mice. **E**. Parasitemia (blue) and numbers of meningeal trypanosomes (green) in infected mice at 11–12 dpi without or with abatacept treatment. Each symbol corresponds to one mouse.

To examine the role of antigen presentation to T cells in trypanosomal meningitis, we treated mice with abatacept (CTLA4Ig, Orencia), which inhibits activation of naive T cells, in part by binding to CD80/86 on antigen-presenting cells, such as dendritic cells, and inhibiting their interaction with the co-stimulatory receptor CD28 on T cells [52]. To image both T cells and dendritic cells, we created hCD2(DsRed)xCD11c(EYFP) mice. These were injected intraperitoneally with abatacept (10 mg/kg) on alternate days from -1 dpi, and the meninges imaged at 11 or 12 dpi. This treatment markedly reduced the numbers of meningeal T cells and dendritic cells at 11–12 dpi, by factors of about ten (Fig 7A, 7B and 7C). The remaining T cells had a significantly reduced displacement rate (Fig 7D) in part as a consequence of a reduction in mean speed from 11.64 ± 0.34 μm/min (mean ± SEM, n = 3 mice) to 5.2 ± 1.2 μm/min (n = 3; *p* = 0.007). However, treatment with abatacept had no evident effect on parasitemia (Fig 7E). Nor was there a conclusive effect on the numbers of trypansomes in the dura: trypanosomes were detected in the meninges of 2 of 6 untreated mice, and in one of 6 treated mice (Fig 7E), Thus abatacept greatly reduced and modified the immune response in the dura, but its effect, if any, on the early appearance of trypanosomes there is unresolved.

### The dendritic cells in the dura made prolonged contacts with T cells, suggesting antigen presentation

**Fig 8:**
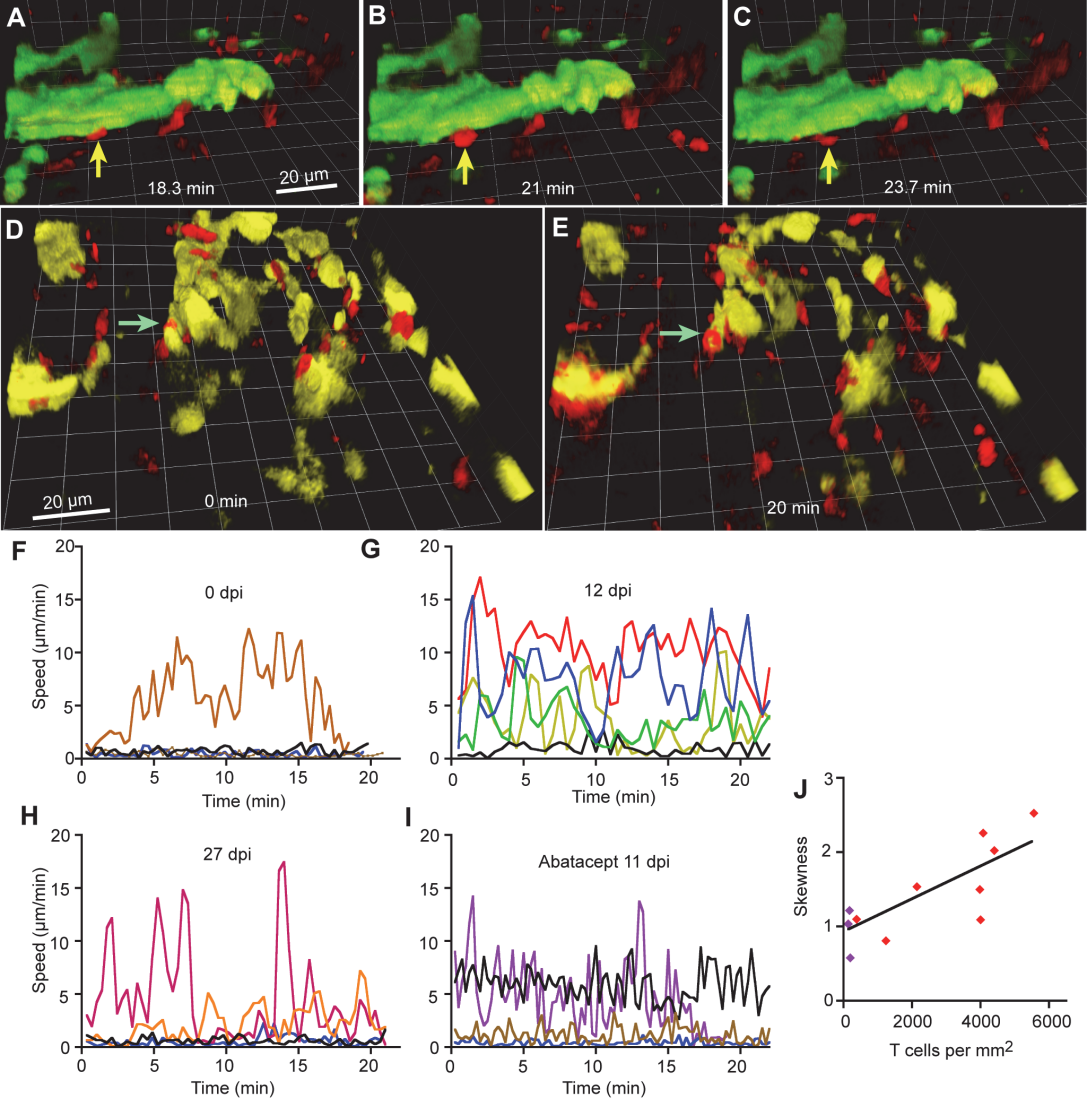
T cells contacted dendritic cells in the meninges. **A-C**. A T cell that made contact with a dendritic cell for 5.4 min. During 25 min of imaging, the T cell indicated by the arrow contacted a dendritic cell (green, part of an agglomeration) at 18.3 min (**A**) remained in place (**B**), and left at 23.7 min (**C**). 25 dpi. **D,E**. A T cell (arrow, red) that remained in contact with a dendritic cell (yellow) throughout 20 min of imaging (S13 Video). 25 dpi. Both scale bars 20 μm. **F-I**. Illustrative plots of T cell speed changing with time. **J**. The mean skewness of the distribution of speed about the mean speed of at least 10 tracks per mouse was calculated and plotted against the number of T cells in the dura. Each symbol corresponds to one mouse. Purple symbols indicate three mice treated with abatacept.

Long-lasting antigen presentation by dendritic cells to T cells, which is inhibited by abatacept, can profoundly modify the phenotype of a developing immune response [53, 54]. In infected hCD2(DsRed) x CD11c(EYFP) mice, visual inspection of videos showed that nearly all contacts lasted less than 2 min, but some longer contacts were observed, as in Fig 8A–8C (6 min) and Fig 8D and 8E (> 20 min, arrows; S13 Video). At least 17 cases of contact lasting more than 15 min were observed (in a total of 155 min of video record). T cells normally respond only to antigens presented on host MHC and in accordance with this rule they were not observed to make contact lasting more than a fraction of a second with trypanosomes (S9 Video).

To quantify the interactions of T cells in large populations, including those in mice without fluorescent dendritic cells, we used automated tracking to plot variations in speed against time for sample T cell tracks. Illustrative plots are shown in Fig 8F–8I. In uninfected mice, the plots confirmed that most cells were stationary and a small number moved (Fig 8F). At early infection (e.g., Fig 8G), more cells were moving. By 27 dpi, some cells were seen to stop for some minutes, then move rapidly (Fig 8H). Treatment with abatacept (which reduced mean speed and displacement rate—Fig 7E) also reduced major changes in speed (Fig 8I). The distribution of instantaneous speeds of cells that briefly moved fast between longer periods of arrest will be positively skewed about the mean, so we calculated the skewness [40] for samples of tracks under various conditions. Skewness was positive, not obviously related to dpi, or to the numbers of dural trypanosomes, but correlated significantly with the number of T cells (Fig 8J; r^2^ = 0.60, slope different from zero with *p* = 0.008). The results for three mice treated with abatacept lay on the same line (purple symbols in Fig 8J). This graph quantifies the idea that when T cells are numerous they move rapidly between attachments, presumably to dendritic cells.

### The sources of the increases in T cells, dendritic cells and trypanosomes in the dura

**Fig 9:**
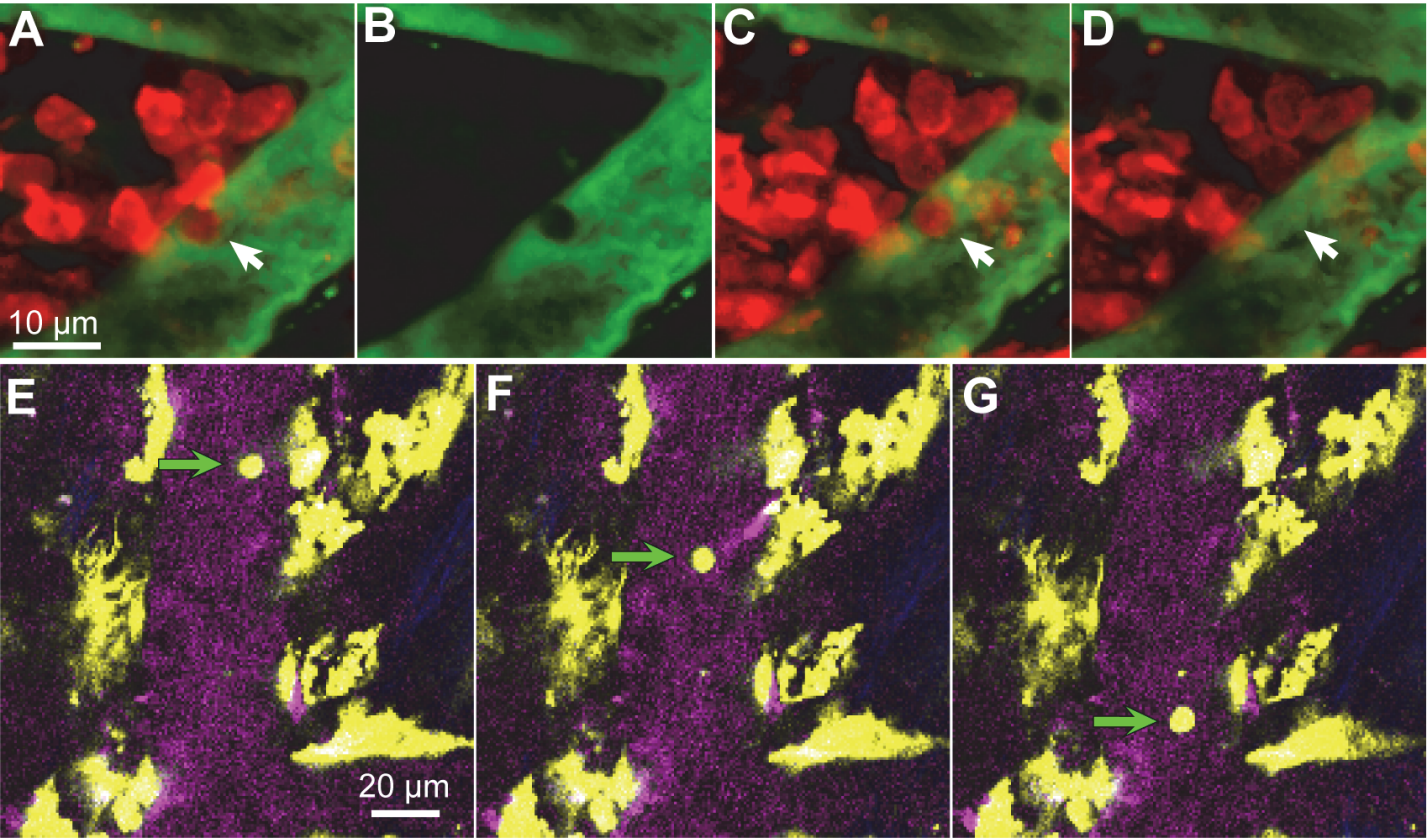
T cells and CD11c+ cells show some interaction with the vascular endothelium. **A.** A T cell (red, arrow) is almost stationary. **B.** The green blood marker image of the same frame confirms that the T cell was intravascular. **C**. 18 min later the T cell has moved only 2.5 μm. **D**. 20s after that it has moved out of the field. **D,E,F.** Frames at 2s intervals show a CD11c+ cell moving slowly (19.3 μm/s) in a blood vessel.

Extravasation of leukocytes normally occurs by diapedesis, which takes several minutes and is preceded by a period of arrest on the vascular endothelium, which may last for hours [21, 55, 56]. Fluorescent T cells and dendritic cells were readily observed in meningeal blood vessels (Fig 9A and 9E). T cells were observed to attach to the vascular endothelium and crawl slowly (Fig 9A–9D). CD11c^+^ cells were not observed to crawl or halt, but, in rare cases, were observed to roll (Fig 9E and 9F). These rolling cells did not have a dendritic shape, and may have been macrophages. For neither cell type did we observe an unambiguous extravasation, but the prolonged interaction sometimes seen between T cells and vascular endothelium suggests that at least some of the extravascular T cells had arrived by classical diapedesis.

**Fig 10:**
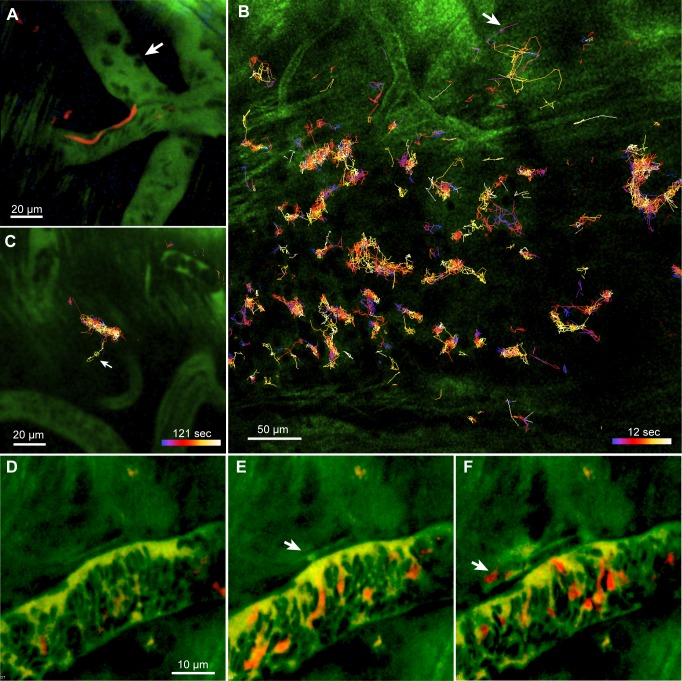
Arrival of trypanosomes in the dura mater. **A**. In dural vessels, trypanosomes were observed to move rapidly (red streaks, S2 Video) while leukocytes (dark, excluding plasma marker) could be arrested (e.g., at arrow). 26 dpi. **B**. Approximate tracks of trypanosomes within the focal depth of an XY scan, acquired over 12 s. A few excursions away from restricted localities are seen (e.g., arrow). Time is color-coded from blue to white (scale). 13 dpi. **C**. At another site in the dura of the same mouse1000 frames were acquired over 121 sec. Arrow indicates a movement out of the generally restricted volume. **D-F**. Frames from S14 Video showing extravasation of a trypanosome. The blood marker (dextran-fluorescein, 70 kDa) appears yellow-green, the trypanosomes express mCherry. In (**E**) blood plasma leaks from the vessel (arrow). 4 sec later, a trypanosome appears outside the vessel (**F**, arrow). Excitation wavelength 1050 nm. Exceptionally, **D**-**F**, are from a CD1 mouse infected with *T*.*b*.*brucei* Lister S427, at 3 dpi.

Previous authors have suggested that trypanosomes arrive in the brain parenchyma either by diapedesis [12, 17] or by transport in CSF from the choroid plexus [57, 58], so we first looked for evidence of similar processes in the dura. Diapedesis of trypanosomes would require interaction lasting at least many seconds with the vascular endothelium, but in 24 C57BL/6 mice (and a further 12 CD-1 mice included in [20]), we never observed a trypanosome slowed or arrested on vascular endothelium (Fig 10A and S2 Video). It is therefore unlikely that trypanosomes extravasated from dural vessels by a diapedesis similar to that of leukocytes.

Concerning transport by CSF, it is known that *Trypanosoma brucei* can appear in CSF, perhaps via the choroid plexus [43, 59–63]. They might therefore be carried into subarachnoid space [43, 57, 64]. However, in the present work, trypanosomes were rare or absent in the subarachnoid space (Fig 4C and 4D) so the hypothesis would require that trypanosomes cross the arachnoid membrane [25] from a (hypothetical) population at a very low density to accumulate, at a higher density, in the dura, and this is unlikely. Nor did the movement of trypanosomes in the dura suggest that they arrived by migration from some source elsewhere in the cranium, in which case, they would migrate across the imaging field. Instead, the trypanosomes in the dura appeared to be mainly confined within localities less than 30 μm across and were never seen to move smoothly as if carried by a flow of fluid (Fig 10B and S4 and S5 Videos). There were occasional abrupt displacements over longer distances (arrow in Fig 10C), but these occurred in all directions. In a sample of 1901 tracks of trypanosomes recorded over up to 121 sec (1000 frames) in XY scans there was no sign of net migration, the mean component of displacement rate in the rostral direction lying within 95% confidence limits of -0.104 and +0.305 μm/sec and in the medial direction of -0.169 and +0.232 μm/sec.

In the inflamed dura, proteins can extravasate [65], presumably by transient, localized, opening of the vascular endothelium [66–69]. Trypanosomiasis is known to increase vascular leakage in the meninges [70, 71]. In the present work, most of our time-series imaging was done with excitation wavelengths chosen to maximize fluorescence from trypanosomes or lymphocytes rather than from the blood marker. However, plasma extravasation was observed at one vascular site (with *T*.*b*.*brucei* Lister 427 in a CD-1 mouse): blood plasma labeled with fluorescent 70 kDa dextran was released over about 4 sec and a second release occurred 16 sec later. Remarkably, the first release of plasma was accompanied by a trypanosome (Fig 10D, 10E and 10F and S14 Video). We did not observe another such extravasation, but we can estimate that the probability of observing one was small. Choosing, conservatively, a case of unusually rapid invasion, we counted 797 trypanosomes per mm^2^ in the dura at 11 dpi (Fig 2B). If the invasion had occurred over, say, 48h, then that is one extravasation in 217 sec per mm^2^. To count trypanosomes we typically imaged, at most, 8 fields of 0.02 mm^2^ for 12 sec each, in each mouse. We can therefore estimate that, on average, and if we used appropriate imaging conditions, we would capture one extravasation for every 113 mice. These arguments suggest that rapid extravasation might account for the arrival of trypanosomes in the dura, and explain why we observed it only once. The lack of correlation between the numbers in the dura and in the blood at any one time (Fig 3F) might be accounted for because the numbers in the dura were the cumulative result of extravasation at times with varying parasitemia.

More than 4,025 extravascular trypanosomes in the meninges of more than 62 mice were videoed and counted (including mice and trypanosomes of several strains), and more were observed but not videoed. No sign of cell division was observed: neither conjoined trypanosomes nor trypanosomes with more than two concentrations of DNA labeled with furamidine (in the nucleus and the kinetoplast).

### Trypanosomes in the dura make shorter displacements as infection progresses

**Fig 11:**
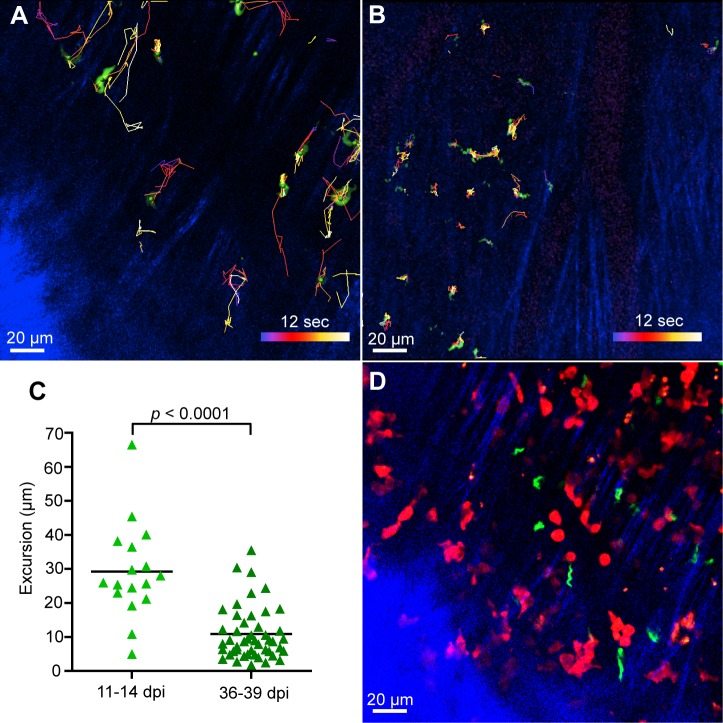
As infection progressed, trypanosomes in the dura made shorter displacements. Automated tracking suggests that at 11 dpi (**A**) extravascular trypanosomes make longer displacements compared to 32 dpi (**B**). Scale bar applies to both images. **C**. Maximum excursions measured on 12s videos. Each symbol is the mean of the maximum excursions for all trypanosomes imaged one image field. Pooled results from 3 mice 11–14 dpi and 2 mice 36 and 39 dpi. **D**. Another frame from the mouse of (**A**) showing trypanosomes and T cells in approximately the same plane, but apparently not interacting (see also S9 Video). All scale bars are 20 μm.

The number of extravascular trypanosomes in the dura appeared to peak after 30 dpi and then fall to zero (Fig 3D). This means that either trypanosomes moved out of the dura, or were destroyed within the dura, at a rate that could exceed the rate of entry. Automated tracking of trypanosomes suggested they displaced less at later infection times (Fig 11A and 11B). To check this, without relying on automated tracking of the rapidly moving trypanosomes, we visually followed their positions on the videos and noted the co-ordinates of the two extreme positions visited by each trypanosome during the standard imaging period of 12 s. This analysis confirmed that the excursions were fewer and shorter at later stages of infection (the mean values being 10.8 μm, SD = 7.7 μm at 36–39 dpi compared to 29.2 μm ± 13.9 μm at 11–14 dpi, *p* < 0.0001; Fig 11C). This progressive reduction in movement, and the decline in the dural population after 30 dpi (Fig 3D), suggest that the dura becomes an unfavorable environment for trypanosomes.

## Discussion

### Lymphocytes in the dura

In rats, experimental autoimmune encephalomyelitis (a model of multiple sclerosis) causes an increase in T cells outside leptomeningeal blood vessels in the exposed spinal cord [21], and injection of adjuvant causes an increase in W3/25^+^ helper T cells in the leptomeningeal layers that adhere to the cortical parenchyma [72]. In a mouse stroke model, observation through the thinned skull revealed T cells outside horizontal, presumably pial, vessels [19]. In contrast, the major increases in the numbers of T cells and CD11c+ cells that we observed in trypanosomiasis were in the dura. Another parasite, the intestinal nematode *Nippostrongylus braziliensis*, is known to inflame the dura, as indicated by an increase in the mast cell population [32]. Since the dura is separated from the leptomeningeal spaces by the impermeable arachnoid membrane ([23–25], Fig 4E) the distinction may have functional significance. The increase in lymphocyte numbers was greatly reduced by abatacept, which suggests that it required antigen presentation to T cells. This agrees with previous results showing that trypanosomiasis leads to activation of peripheral T cells [5–8]. It is also known that, in trypanosomiasis, lymphocytes can appear in the meninges of the basal brain [42], in perivascular space [42] and in the CSF [59, 73]. We did indeed observe small numbers of T cells in spaces filled with CSF: beside pial vessels (Fig 5E) or in the perivascular space of descending vessels (Fig 5F), as well as the much greater numbers in the dura.

Interactions lasting several minutes were observed between CD11c+ cells and T cells in the dura, suggesting that antigen presentation to T cells also took place in this site. This is supported by the reduction in the frequency of halts made by T cells in the dura after abatacept treatment (Fig 8I). The data are compatible with antigen presentation in the dura occurring only after trypanosomes were present, but do not exclude the contrary.

The dura is an interface between the nervous system and the immune system in that peptides released from efferent endings of the trigeminal nerve stimulate mast cells to release mediators, including substance P and histamine, which in turn activate afferent pain fibers [32, 74]. Whether immune activity in the dura sends signals to the CSF appears to be unknown: conceivably, cytokines or chemokines might cross the arachnoid membrane, or arachnoid cells might be stimulated to release signaling molecules.

### Trypanosomes in the dura

Because we avoided making a craniotomy, the structure of the meninges was intact and we were able to locate the trypanosomes in the cortical dura. We were unable to confirm the observation of early invasion of the superficial parenchyma made by Frevert et al.[45] in craniotomized mice, except in one case, when the skull was accidentally penetrated. The blood vessels of the dura, like those of the choroid plexus, are relatively leaky compared to those of the subarachnoid space and most of the parenchyma [24, 75] and it seems unsurprising that both should be sites of early extravasation of trypanosomes [42, 43, 76, 77]. Drug treatments that kill trypanosomes in the periphery, but not in the brain parenchyma, kill trypanosomes in the meninges [20], in the compartment we now identify as the dura.

The number of trypanosomes in the dura of C57BL/6 mice did not continually increase with dpi, and appeared to fall between 30 and 40 dpi. Similarly, in CD-1 mice, low counts in the dura could be found at the later times [20]. Hence the balance of influx of trypanosomes into the dura and their removal, either by emigration or destruction, can shift towards removal. We did not find extravascular trypanosomes below the dura, so have no evidence of migration downwards towards the parenchyma. The arachnoid membrane, with its tight junctions, is expected to be a greater obstacle to trypanosome passage than, for example, the weak barriers between circumventricular organs and blood or CSF [78]. It therefore seems unlikely that trypanosome invasion spreads from the dura to the parenchyma. We did not see evident movement of trypanosomes along dural lymph ducts [30], and we cannot comment on the possibility that trypanosomes moved back into the blood. However, the observations appear to be compatible with the hypothesis that trypanosomes in the dura are destroyed, this destruction occurring at a time when the dura is densely packed with T cells and CD11c+ cells and the extravascular trypanosomes are moving shorter distances (Fig 11C). Likely contributors to this debilitation are cytokines such as interferon gamma [79], prostaglandin D_2_ produced by trypanosomes [80] or neuropeptides from nerve endings or mast cells [81]. Another possibility is impaired energy metabolism. Trypanosomes and activated lymphocytes are crowded into the dural space and both produce ATP mainly by glycolysis, which requires rapid consumption of glucose [82–85] and, by producing pyruvic [86] or lactic acid, could acidify extracellular fluid [87–90]. The red fluorescence sometimes seen in the nucleus and kinetoplast after furamidine injection (Fig 3C and 3D) has not been reported in vitro, and might, perhaps, reflect an aberrant pH. A contrary possibility is that physical restriction of movement in the crowded environment of the dura is harmful to trypanosomes. In vitro, simply immobilizing trypanosomes can kill them [91], and it has been argued that, in vivo, swimming protects trypanosomes against antibodies. Antibodies attached to a freely swimming trypanosome are swept from the forward-pointing flagellum into the flagellar pocket and endocytosed [92] and certain mutations that impair flagellar function [92, 93], but not all [94], are lethal.

### Methodological point

Imaging through the calvaria was initially developed to study the neural brain (e.g. [37]). Apart from the intrinsic interest of the cortical meninges in many diseases, it is technically a convenient place to study lymphocyte dynamics because the tissue is held rigidly by the skull, is approximately two-dimensional, and can be accessed optically without apparent damage.

## Conclusion

In vivo imaging through the skull made it possible to localize and video accumulations of T cells, dendritic cells and trypanosomes in the cortical dura. Apart from using abatacept to show the requirement for T cell activation, we have confined ourselves in the present paper to describing the numbers and movements of these actors. Numerous questions are raised, including how (or if) infiltration of the dura by lymphocytes and trypanosomes contributes to neuropathology, and whether there is similar lymphocyte behavior in the dura in meningitis caused by other pathogens.

## Supporting Information

S1 Fig The mouse holder. A scale drawing of the base plate to which the mouse was attached by a skull plate. Most of the machined parts were of brass (stainless steel or PTFE would have been better).
(PDF)

S2 Fig Delivery of heating, anesthetic, and superfusate for the skull. **A.** Thinning the skull. **B.** The mouse under the two-photon microscope.
(PNG)

S3 Fig Skull bone shows second harmonic generation (SHG). **A** and **B** show the same field with excitation at 820 nm in **A** and 1050 nm in **B**. Emission < 490 nm is shown as blue and > 495 nm as green. This result shows that the emission is SHG rather than fluorescence.
(PDF)

S4 Fig Labeling of host cells by intravenous furamidine. (PDF)

S5 Fig Limited migration of T cells. Plots of mean velocities, (**A**) in the X and Y plane and (**B**) in the Z direction.
(PDF)

S1 Video Time-lapse video of CD11c^+^ (EYFP) dendritic cells in the meninges of an uninfected mouse. Scale bar 38 μm. Imaged through the skull with excitation wavelength 960 nm.
(MOV)

S2 Video Real-time video of intravascular and extravascular fluorescent trypanosomes. Fast-moving intravascular trypanosomes appear as red streaks. Some leukocytes (visible by exclusion of green blood marker) are seen to be arrested. 26 dpi. It may be necessary to open this video from the Quick Time Player application.
(MOV)

S3 Video mCherry trypanosomes in ventral brain in an ex-vivo brain slice. Shows trypanosomes expressing mCherry and host cell nuclei (blue) previously labeled by intravenous injection of furamidine. 36 dpi. Frame width 212 μm. 2.56 frames/s. Simultaneous excitation at 800 and 1040 nm. The mouse had been perfused through the heart and 1 mm slices cut and superfused with glucose-containing saline. This is the only ex vivo video in this paper.
(MOV)

S4 Video *T*.*b*.*brucei* GVR35 GFP-expressing trypanosomes (green) in the cortical dura mater, imaged through the thinned skull in vivo. Collagen fibers appear blue, blood vessels show faint magenta labeling. 32 dpi. Width of frame 212 μm, 8.3 frames/s, anterior upwards, left lateral to left. The microscope scanned a single XY plane, but excited fluorescence over a depth > 5 μm. Excitation wavelength 864 nm. Collagen SHG detected at <490 nm.
(MOV)

S5 Video Trypanosomes in the dura in vivo, labeled by a previous intravenous injection of furamidine. Excitation wavelength 780 nm. Host nuclei have blue fluorescence, trypanosome nuclei and kinetoplasts showed blue or, as here, red fluorescence (wavelength > 555 nm).
(MOV)

S6 Video A GFP trypanosome struggling through collagen just below the skull. 27 dpi. Frame width 110 μm. Excitation wavelength 940 nm. It may be necessary to open this video from the Quick Time Player application.
(MOV)

S7 Video mCherry trypanosomes moving close to small dural blood vessels. 21 dpi. Frame width 212 μm. Excitation wavelength 1040 nm, SHG shown as green, blood marker 705 nm quantum dots. 21 dpi.
(MOV)

S8 Video A T cell apparently squeezing between collagen fibers. 11 dpi. Frame width 212 μm. It may be necessary to open this video from the Quick Time Player application.
(MOV)

S9 Video T cells and trypanosomes moving in the same XY plane. GFP trypanosomes, DsRed T cells. Frame width 212 μm. 11 dpi. From the same mouse as Fig 11A and 11D.
(MOV)

S10 Video T cell movements in an uninfected mouse. Frame width 345 μm. One moving T cell, 2 stationary. Scale bar 50 μm. It may be necessary to open this video from the Quick Time Player application.
(MOV)

S11 Video T cell movements at 27 dpi: perspective view. It may be necessary to open this video from the Quick Time Player application.
(MOV)

S12 Video T cell movements at 40 dpi: side view with tracks. (MOV)

S13 Video A T cell remaining in contact with a dendritic cell throughout 20 min of imaging. See Fig 9D and 9E for the site of the contact. 25 dpi, T cells express DsRed, dendritic cells express EYFP, excitation wavelength 987 nm. The grid spacing is 14.2 μm. It may be necessary to open this video from the Quick Time Player application.
(MOV)

S14 Video Abrupt extravasation of blood marker (dextran-fluorescein, green). The first of two extravasations was accompanied by a trypanosome (red). Width of frame 50 μm. It may be necessary to open this video from the Quick Time Player application.
(MOV)
