# Supplementary figures and images for: Intravital Imaging of a Massive Lymphocyte Response in the Cortical Dura of Mice after Peripheral Infection by Trypanosomes
Source: PLoS Negl Trop Dis. 2015 Apr 16;9(4):e0003714. doi: 10.1371/journal.pntd.0003714 (PMC4400075; doi:10.1371/journal.pntd.0003714)

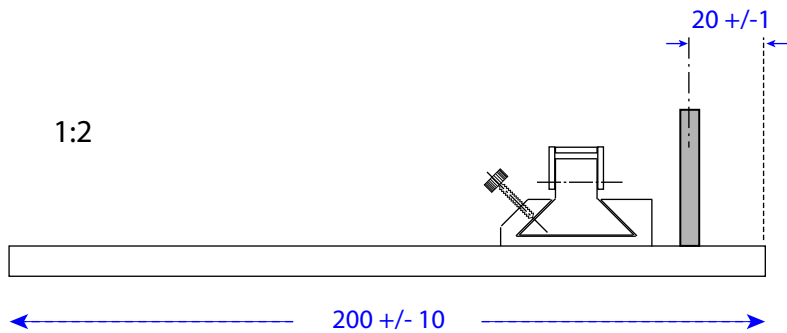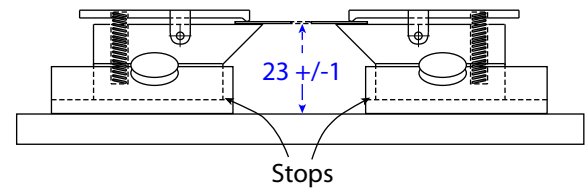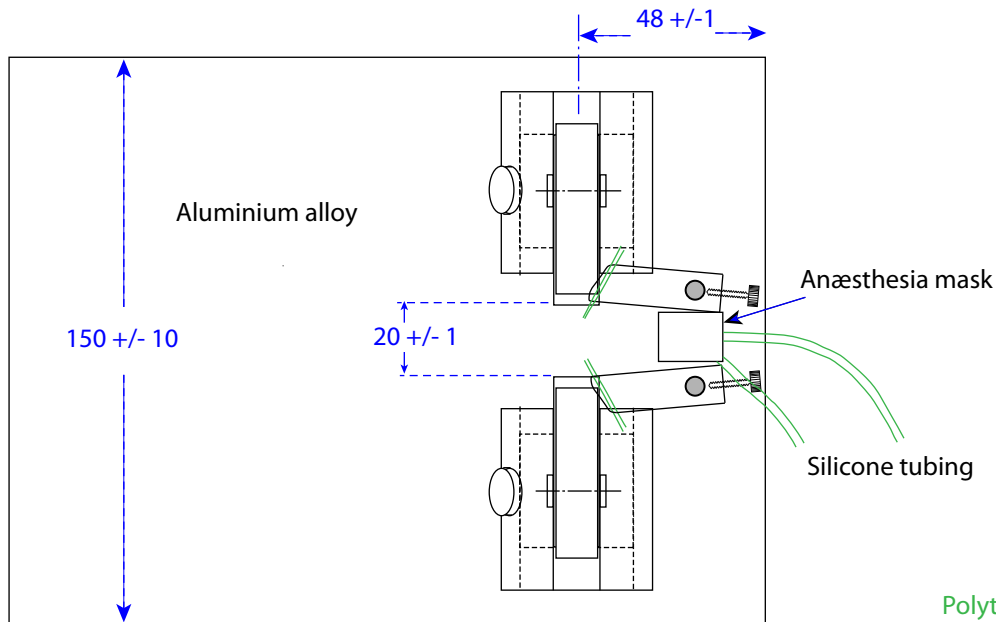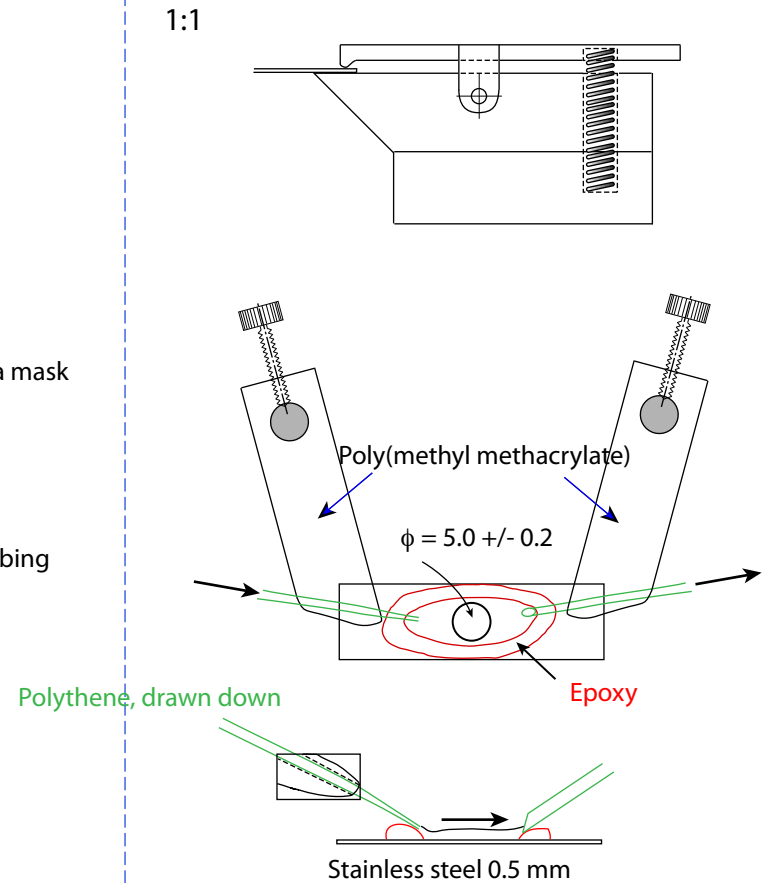

Supplement: S1 Fig — A scale drawing of the base plate to which the mouse was attached by a skull plate. Most of the machined parts were of brass (stainless steel or PTFE would have been better). (PDF) [file pntd.0003714.s001.pdf]

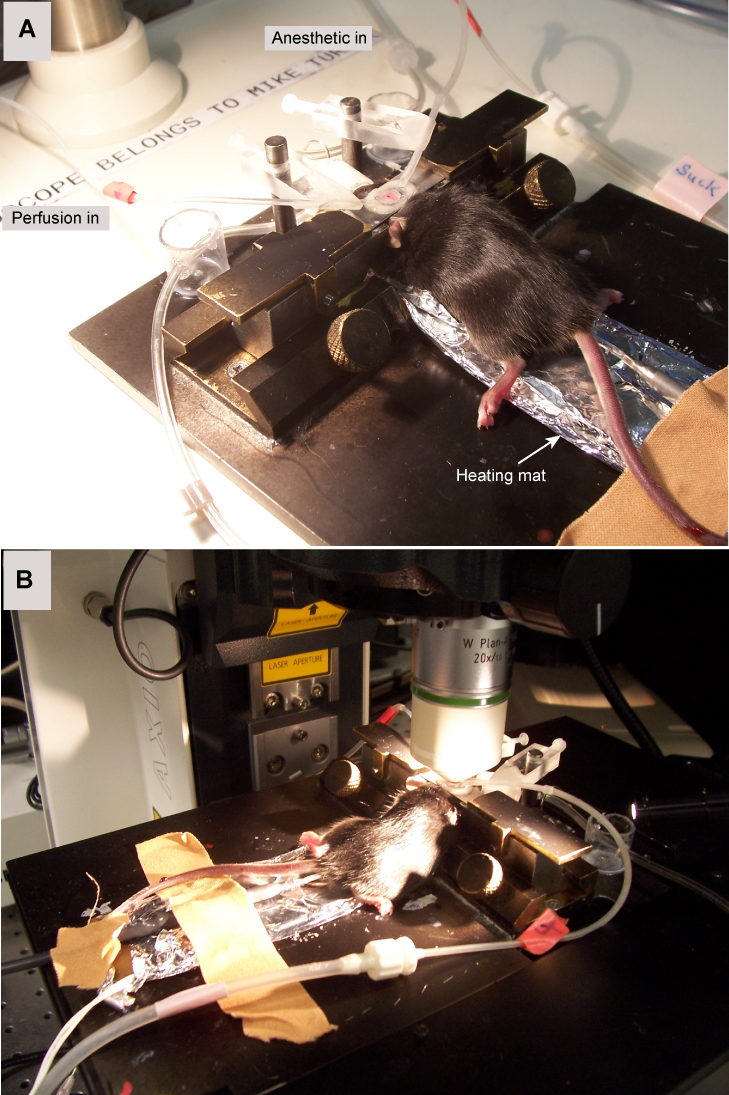

Supplement: S2 Fig — A. Thinning the skull. B. The mouse under the two-photon microscope. (PNG) [file pntd.0003714.s002.png]

**A**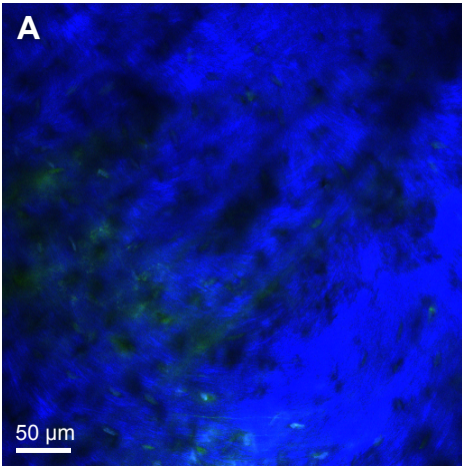**B**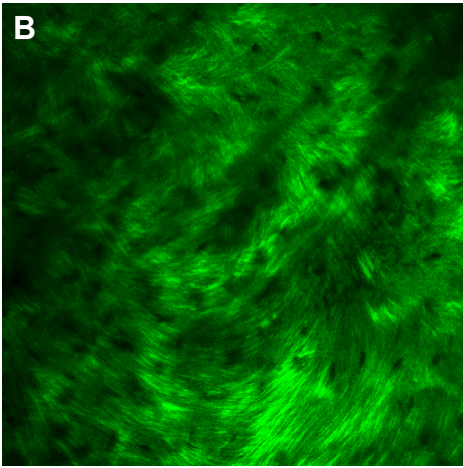

Supplement: S3 Fig — A and B show the same field with excitation at 820 nm in A and 1050 nm in B. Emission < 490 nm is shown as blue and > 495 nm as green. This result shows that the emission is SHG rather than fluorescence. (PDF) [file pntd.0003714.s003.pdf]
