# Supplementary material for: Intravital Imaging of a Massive Lymphocyte Response in the Cortical Dura of Mice after Peripheral Infection by Trypanosomes
Source: PLoS Negl Trop Dis. 2015 Apr 16;9(4):e0003714. doi: 10.1371/journal.pntd.0003714 (PMC4400075; doi:10.1371/journal.pntd.0003714)
Supplement: S4 Fig — (PDF) [file pntd.0003714.s004.pdf]

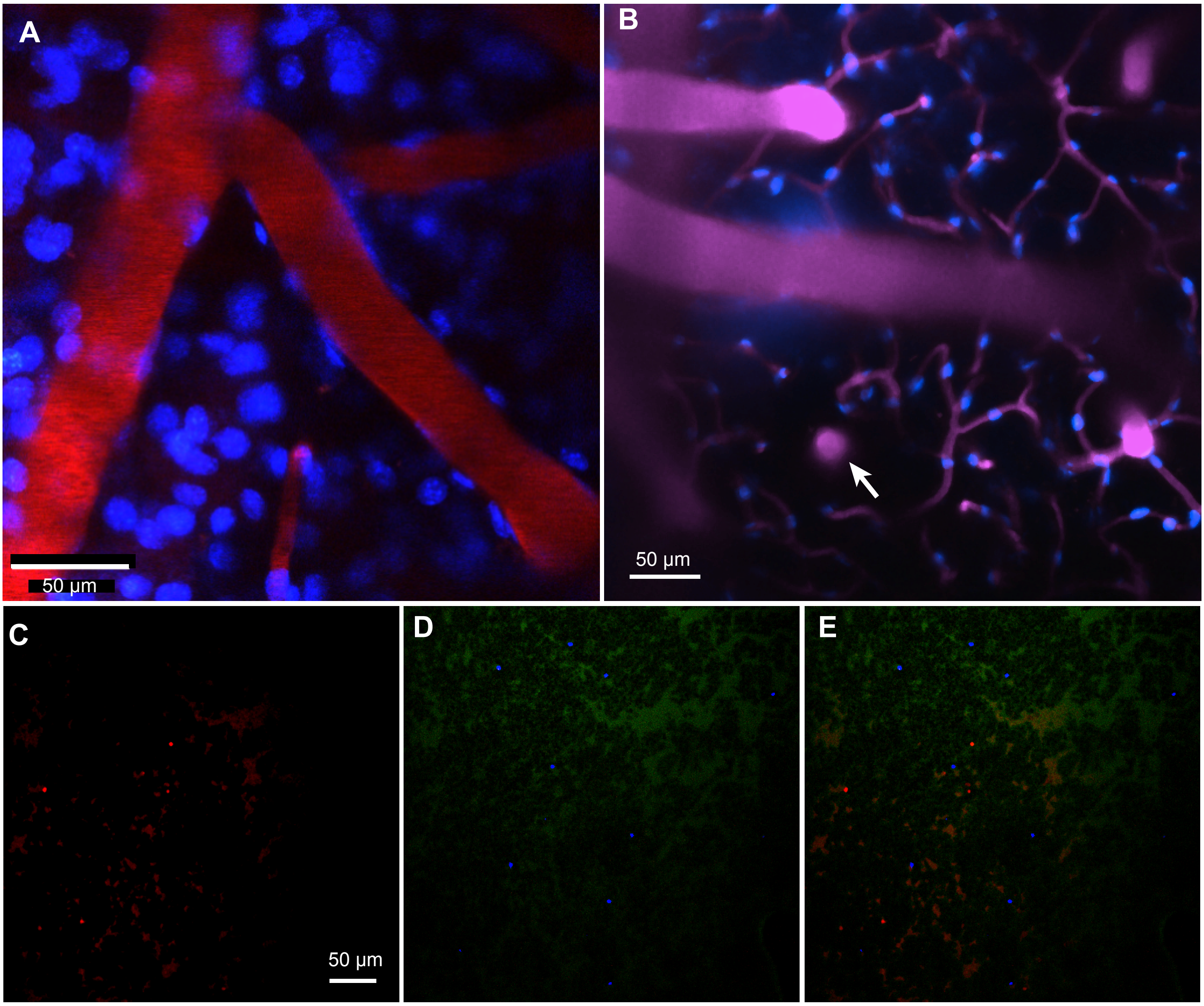

S4 Figure. Labeling of host cells by intravenous furamidine. **A,B**. Two-photon imaging through the thinned skull. **A**. Blue fluorescence from the nuclei of dural cells and of endothelial cells of pial vessels.
B. A Z-projection 100 µm deep extending from large horizontal pial vessels into the parenchyma with its vertical vessels (arrow) and sinuous capillaries. Nuclei of vascular endothelial cells are labeled. **C-D**. A blood smear from a reporter mouse expressing DsRed in $CD2^{+}$ T lymphocytes. **C**. DsRed. **D**. Furamidine. **E**. Merged image showing that unidentified leukocytes that are not T lymphocytes are labeled by furamidine.
