# Supplementary material for: Intravital Imaging of a Massive Lymphocyte Response in the Cortical Dura of Mice after Peripheral Infection by Trypanosomes
Source: PLoS Negl Trop Dis. 2015 Apr 16;9(4):e0003714. doi: 10.1371/journal.pntd.0003714 (PMC4400075; doi:10.1371/journal.pntd.0003714)
Supplement: S5 Fig — Plots of mean velocities, (A) in the X and Y plane and (B) in the Z direction. (PDF) [file pntd.0003714.s005.pdf]

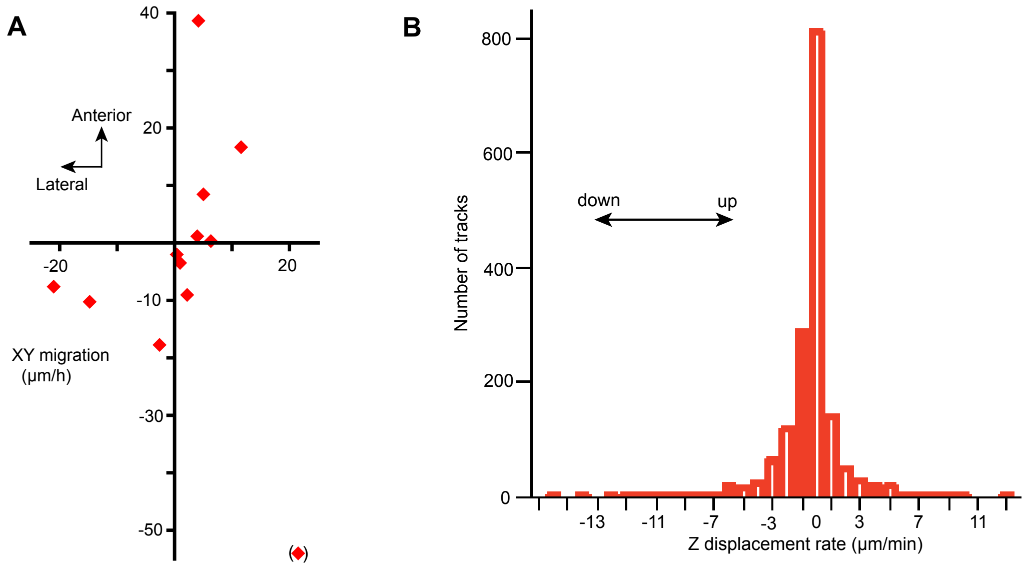

S5 Fig. Limited displacement by T cells in the meninges. A. The X (medial) and Y (anterior) components of (vector displacement/step duration) were averaged for all measured tracks in each mouse. Each point corresponds to one mouse. The outlying value (bracketed) was dominated by one very mobile cell among a small total number. **B**. The Z vector of the displacement rate for 2,461 tracks from 4 mice, 10 - 30 dpi was calculated and the results shown as a frequency histogram. Note the different speed units in **A** and **B.**
